# Abnormal Spontaneous Brain Activities of Limbic-Cortical Circuits in Patients With Dry Eye Disease

**DOI:** 10.3389/fnhum.2020.574758

**Published:** 2020-11-09

**Authors:** Haohao Yan, Xiaoxiao Shan, Shubao Wei, Feng Liu, Wenmei Li, Yiwu Lei, Wenbin Guo, Shuguang Luo

**Affiliations:** ^1^Department of Psychiatry, National Clinical Research Center for Mental Disorders, The Second Xiangya Hospital of Central South University, Changsha, China; ^2^Department of Neurology, The First Affiliated Hospital of Guangxi Medical University, Nanning, China; ^3^Department of Radiology, Tianjin Medical University General Hospital, Tianjin, China; ^4^Department of Psychiatry, The Third People's Hospital of Foshan, Foshan, China

**Keywords:** dry eye disease, resting-state functional magnetic resonance imaging, regional homogeneity, support vector machine, limbic-cortical circuits

## Abstract

Whether brain function is altered in patients with dry eye disease (DED) remains unclear. Twenty patients with DED and 23 healthy controls (HCs) were scanned using resting-state functional magnetic resonance imaging. Regional homogeneity (ReHo) and support vector machine (SVM) were used to analyze the imaging data. Relative to the HCs, the patients with DED showed significantly increased ReHo values in the left inferior occipital gyrus (IOG), left superior temporal gyrus, and right superior medial prefrontal cortex, and significantly decreased ReHo values in the right superior frontal gyrus/middle frontal gyrus and bilateral middle cingulum (MC). SVM results indicated that the combination of ReHo values in the left MC and the left IOG in distinguishing patients with DED from HCs had a sensitivity of 95.00%, a specificity of 91.30%, and an accuracy of 93.02%. The present study found that the patients with DED had abnormal ReHo values in the limbic-cortical circuits. A combination of ReHo values in the left MC and the left IOG could be applied as a potential imaging biomarker to distinguish patients with DED from HCs. The dysfunction of limbic-cortical circuits may play an important role in the pathophysiology of DED.

## Introduction

Dry eye disease (DED) is a common health problem because of its morbidity, prevalence (5–35%), and cost implications (Smith, 2007; Messmer, 2015). DED is more frequently reported by women than by men, and its likelihood increases with age (Moss et al., 2000; Schaumberg et al., 2003). Dry eye (DE) is defined as “a multifactorial disease of the ocular surface characterized by a loss of homeostasis of the tear film accompanied by ocular symptoms, in which tear film instability and hyperosmolarity, ocular surface inflammation and damage, and neurosensory abnormalities play etiological roles” by the Tear Film and Ocular Surface Society Dry Eye Workshop II (Craig et al., 2017). “Neurosensory abnormalities” are indeed included in the DE definition, but the neuropathophysiology mechanism of DED remains unclear.

The tear film plays an important role in providing a refractive interface for the optical light path, and protection and lubrication for the ocular surface (Willcox et al., 2017). The major ingredients of the tear film are aqueous tear, lipid components, and mucins. Aqueous tear is mainly produced by lacrimal glands. Lipid components are generally synthesized by meibomian glands. Mucins are mainly synthesized by conjunctival goblet cells (Ellingham et al., 1999) and stratified squamous epithelial cells (Argüeso et al., 2003). DED may be caused by the dysfunction of these glands and cells (Mathers, 2000; Mantelli and Argüeso, 2008) or the neuronal circuits regulating tear secretion (van Bijsterveld et al., 2003; Dartt, 2009). In DED, aberrations and scattering induced by alterations of the tear film directly cause disturbances in vision quality (Benito et al., 2011; Tan et al., 2015). In addition to visual disturbance, most patients with DED also complain of ocular discomfort accompanied by increased blinks. Ocular discomfort comprises ocular fatigue and unpleasant sensations such as pain, itching, and dryness. The unpleasant sensations are induced by the pathological processes affecting the trigeminal sensory nerves innervating the ocular and periocular tissues. In some studies, the terms “dryness,” “itching,” “foreign body sensation,” and “burning” are applied to describe the ocular pain associated with DED (Mertzanis et al., 2005; Kalangara et al., 2016). Ocular pain is an uncomfortable and unpleasant sensory and emotional experience induced by the interconnected peripheral nervous system (PNS) and central nervous system (CNS). The enhanced excitability of central pain pathways result from the local activation of microglia and weakened inhibitory descending modulation (Tulleuda et al., 2011). The inhibitory descending control systems come from higher brain centers and modulate ascending excitatory nociceptive pathways by influencing the trigeminal and spinal sensory input (Tracey and Mantyh, 2007; Khasabov et al., 2015). However, the role of descending control systems in DED has not been comprehensively studied. Ocular pain in patients with DED is mainly induced by peripheral insults to the innervated ocular and periocular tissues. However, in certain circumstances, ocular pain in patients with DED is induced by direct injuries to, or dysfunctions of, the cortical and subcortical structures, which process the peripheral nociceptive input, the peripheral nociceptive sensory neurons located in the trigeminal and dorsal root ganglion, and the higher-order neurons located in the spinal cord, brain stem, and thalamus (von Hehn et al., 2012; Belmonte et al., 2015). This pain is called “neuropathic pain.” An emerging concern is that a subset of DED should be represented as a chronic neuropathic pain (Kalangara et al., 2016). In DED, the persistent deficiency of tears results in peripheral nerve damage and ocular inflammation. Peripheral nerve damage and ocular inflammation have complex interactions (Ordovas-Montanes et al., 2015). Long-term peripheral nerve damage and ocular inflammation induce alterations in the structures and functions of PNS and CNS involved in ocular sensory pathways (Belmonte et al., 2015, 2017; Rahman et al., 2015; Levitt et al., 2017), thereby leading to neuropathic pain and central sensitization. Central sensitization is caused by decreased activation thresholds and abnormal amplifying signals within the CNS through neuroplastic processes (Latremoliere and Woolf, 2009; Galor et al., 2015). In DED, the increased blinks result from the enhanced activities of the ocular surface sensory nerve evoked by the stimulation of an unstable tear film (Nakamori et al., 1997) and/or from the redistribution of the tear film over the cornea to obtain enhanced image quality (Tan et al., 2015). The level of dopamine released by the basal ganglia can modulate the blink rate in DED, as observed in a rat model study (Kaminer et al., 2011).

In sum, one can reasonably hypothesize that abnormal brain function plays an important role in DED symptoms' maintenance and development. Abnormal brain structure and function in Sjögren syndrome, a subset of DED (Craig et al., 2017), have been reported in many neuroimaging studies. A study applied computed tomography and magnetic resonance imaging (MRI) and identified 24 patients with white matter abnormalities from a total of 49 patients with Sjögren syndrome with DE (Akasbi et al., 2012). White matter hyperintensities were also found in patients with Sjögren syndrome in the studies that applied MRI and voxel-based morphometry (Tzarouchi et al., 2011, 2014). In a study that applied voxel-wise and global brain volume analyses, the patients with Sjögren syndrome showed lower white matter volumes, not gray matter volumes, than the healthy controls (HCs) (Lauvsnes et al., 2014). Another study found decreased gray matter volume in the cortex and cerebellum in patients with Sjögren syndrome (Tzarouchi et al., 2011). A study applied resting-state functional magnetic resonance imaging (rs-fMRI) and functional connectivity analysis and found altered hippocampal functional connectivity in patients with primary Sjögren syndrome (Zhang et al., 2020). Another study applied rs-fMRI and regional homogeneity (ReHo) analysis and found abnormal ReHo values in the frontoparietal junction area and visual cortex in patients with Sjögren syndrome (Xing et al., 2018). Nevertheless, whether brain function is altered in DED remains unclear. Therefore, a study on brain function will facilitate the understanding of the underlying neuropathophysiology of DED.

Herein, we applied rs-fMRI to map functional brain and decipher spontaneous cerebral neuro-activities. Since Bharat Biswal et al.'s study using rs-fMRI (Biswal et al., 1995), rs-fMRI has been widely used to map functional brain and decipher spontaneous cerebral neuro-activities by measuring the blood oxygen level-dependent (BOLD) signal. Relative to task-based fMRI, rs-fMRI can observe cerebral neurophysiological processes without requiring task performance. Therefore, applying rs-fMRI avoids the potential limitation of applying task-based fMRI in fMRI studies.

After accessing the neuroimaging data of patients with DED and HCs via rs-fMRI, ReHo was utilized to depict the local features of BOLD signals and thus reflect the local synchronization of spontaneous brain activities. Kendall's coefficient concordance (KCC) of the voxel similarity of the time series of a given voxel with the nearest neighboring voxels was applied to measure the ReHo values (Zang et al., 2004). ReHo has been successfully utilized to explore the abnormalities of regional functional synchronization in some ophthalmologic illnesses, such as glaucoma, amblyopia, and corneal ulcer (Lin et al., 2012; Chen et al., 2017; Xu et al., 2019). However, whether patients with DED have abnormal ReHo in certain brain regions, particularly in the brain sensory and visual processing regions, remains unclear.

Support vector machine (SVM) learning is a robust classification tool. This supervised learning algorithm is usually used to recognize patterns and analyze data. Relative to Bayesian networks, decision trees, and artificial neural networks, SVM has significant strengths, such as high accuracy, direct geometric interpretation, and excellent mathematical tractability (Zhang and Wu, 2012). Moreover, SVM does not need large training samples to avoid overfitting (Li et al., 2010); this feature is particularly effective in classification when the sample size is small (Chen and Chen, 2017). Therefore, SVM was applied in this work to determine whether abnormal ReHo values in certain brain areas could be used to distinguish patients with DED from HCs.

We hypothesized that patients with DED would show abnormal ReHo in certain brain regions, particularly in the brain sensory and visual processing regions, and that such abnormal ReHo in relevant regions might serve as possible imaging biomarkers to distinguish patients with DED from HCs via SVM.

## Materials and Methods

### Participants

A total of 20 right-handed patients with DED (age ≥18 years) were eligible to participate in the whole study. The diagnosis of DED was confirmed by an ophthalmologist by using DED diagnostic guidelines published in 2007 by the Dry Eye Workshop (Lemp, 2007). The exclusion criteria were as follows: (1) patients with other ophthalmic diseases, such as glaucoma, cataract, diabetic retinopathy, and amblyopia; (2) patients with a history of metabolic encephalopathy, hypertensive encephalopathy, CNS infection, and CNS lesions induced by other causes; and (3) patients who were pregnant.

We recruited 23 right-handed HCs (age ≥18 years) from the community. The sex ratio, age, and years of education of the HCs and patients were group-matched. They were also interviewed using the DED diagnostic guidelines published in 2007 by the Dry Eye Workshop. They were excluded if they had a history of neuropsychiatric illness or brain injury or if they were pregnant.

The ethics committee of the First Affiliated Hospital of Guangxi Medical University approved the study. The study was executed according to the Helsinki Declaration. All participants provided an informed written consent.

### Scan Acquisition

In this study, rs-fMRI was performed using a Siemens 3.0 T scanner and a standard head coil. The participants lay on the scanner bed with their eyes closed. They were instructed to remain calm and awake. They used foam pads and soft earplugs to reduce head motion and scanning noise. A gradient-echo-planar imaging (EPI) sequence was used to acquire the imaging data with the following parameters: repetition time = 2,000 ms, echo time = 30 ms, 30 slices, 90° flip angle, 64 × 64 matrix, 240 mm field of view, 4 mm slice thickness, 0.4 mm gap, and 250 volumes (500 s).

### Data Preprocessing

Statistical parametric mapping software (SPM12; http://www.fil.ion.ucl.ac.uk/spm/) and the Data Processing Assistant for Resting-State fMRI were applied to preprocess the images. The first 10 images of each participant were discarded due to unstable initial MRI signals. We corrected the fMRI images for head motion and acquisition delay between slices. The participants must have translations of <2 mm and rotations of <2° in the x, y, or z axis. Thereafter, all imaging data were spatially normalized to the standard Montreal Neurological Institute (MNI) EPI template in SPM12 and sampled again to 3 mm cubic voxels. The resulting fMRI images were subjected to bandpass filtering (0.01–0.08 Hz) and time-course linear detrending to reduce the influence of high-frequency physiological noise and low-frequency drift.

### ReHo Analysis

ReHo is an rs-fMRI measurement that is utilized to explore regional functional synchronization. The Resting-State fMRI Data Analysis Toolkit (REST, http://resting-fmri.sourceforge.net) was utilized to conduct the ReHo analysis. Individual ReHo maps were produced by calculating the KCC of the time series of a given voxel with those of its nearest voxels (26 voxels). The formula for calculating the KCC value was introduced in a previous study (Zang et al., 2004). The ReHo maps were normalized to reduce the influence of individual variations on the KCC values by dividing the KCC values among each voxel by the whole brain average KCC. Thereafter, the ReHo maps were smoothed with a Gaussian kernel of 4 mm full width at half maximum.

### Statistical Analysis

SPSS 18.0 (Chicago, IL) was used to compare the demographic characteristics of the two groups. To assess the differences in sex distribution, we performed a chi-square test on the two groups. For the differences in ages and years of education, we performed two-sample *t*-tests. The differences in the ReHo values of the HCs and patients with DED were compared by two-sample *t*-tests with years of education and age as the covariates of no interest. The Gaussian random field method was utilized to correct for multiple comparisons with the REST software (voxel significance: *P* < 0.001, cluster significance: *P* < 0.05).

### Correlation Analysis

To assess the possible relationship between abnormal ReHo values and illness duration in patients with DED, we conducted Pearson's correlation analyses between the abnormal ReHo values and illness duration (significance level: *P* < 0.05). In the correlation analysis, the average values of the clusters with abnormal ReHo were used.

### Classification Analysis

Classification analysis was performed to examine whether ReHo values and a combination of ReHo values in relevant regions could serve as possible imaging biomarkers to distinguish patients with DED from HCs. SVM analysis was applied to the classification analysis by using the LIBSVM software package (http://www.csie.ntu.edu.tw/~cjlin/libsvm/) in MATLAB. SVM was performed to measure the capacity of the abnormal ReHo values to distinguish patients with DED from HCs. In SVM, differences between groups are learned by a training dataset, and classification performance in unobserved data is evaluated by a test dataset. To train data, we provided label pairs (*x*_*i*_, *c*_*i*_), *i* = 1, …, *l*. In the label pairs, $x_{i}\in R^{n}$, with *x*_*i*_ representing ReHo values and *c*_*i*_ represents class label. Class label “*c* = +1” was assigned to the patients with DED, and “*c* = 0” was assigned to the HCs. We applied Gaussian radial basis function kernel and Grid search method to implement parameter optimization. We also conducted a “leave-one-out” cross-validation approach in the LIBSVM software to obtain the highest specificity and sensitivity. The form of radial basis function kernel used in the present study is “$K\left(x_{i},x_{j}\right)=e^{-y||x_{i}-x_{j}||2}$” (Liu et al., 2018).

## Results

### Characteristics of Participants

A total of 20 patients with DED and 23 HCs were recruited in the study. The two groups did not show significant differences in sex ratio (*P* = 0.19), age (*P* = 0.23) or years of education (*P* = 0.10). The detailed characteristics of the participants are shown in Table 1.

**Table 1:** Characteristics of participants.

**Variables**	**Patients (*n* = 20)**	**Controls (*n* = 23)**	***p*-value**
Age (years)	52.55 ± 8.66	49.70 ± 6.51	0.23^b^
Sex (male/female)	7/13	4/19	0.19^a^
Years of education (years)	10.20 ± 3.56	8.61 ± 2.27	0.10^b^
Illness duration (months)	20.75 ± 15.37		

a *The p-value for sex distribution was obtained by a chi-square test*.

b *The p-values were obtained by two samples t-tests*.

### Differences in ReHo Values of Patients With DED and HCs

**Figure 1:**
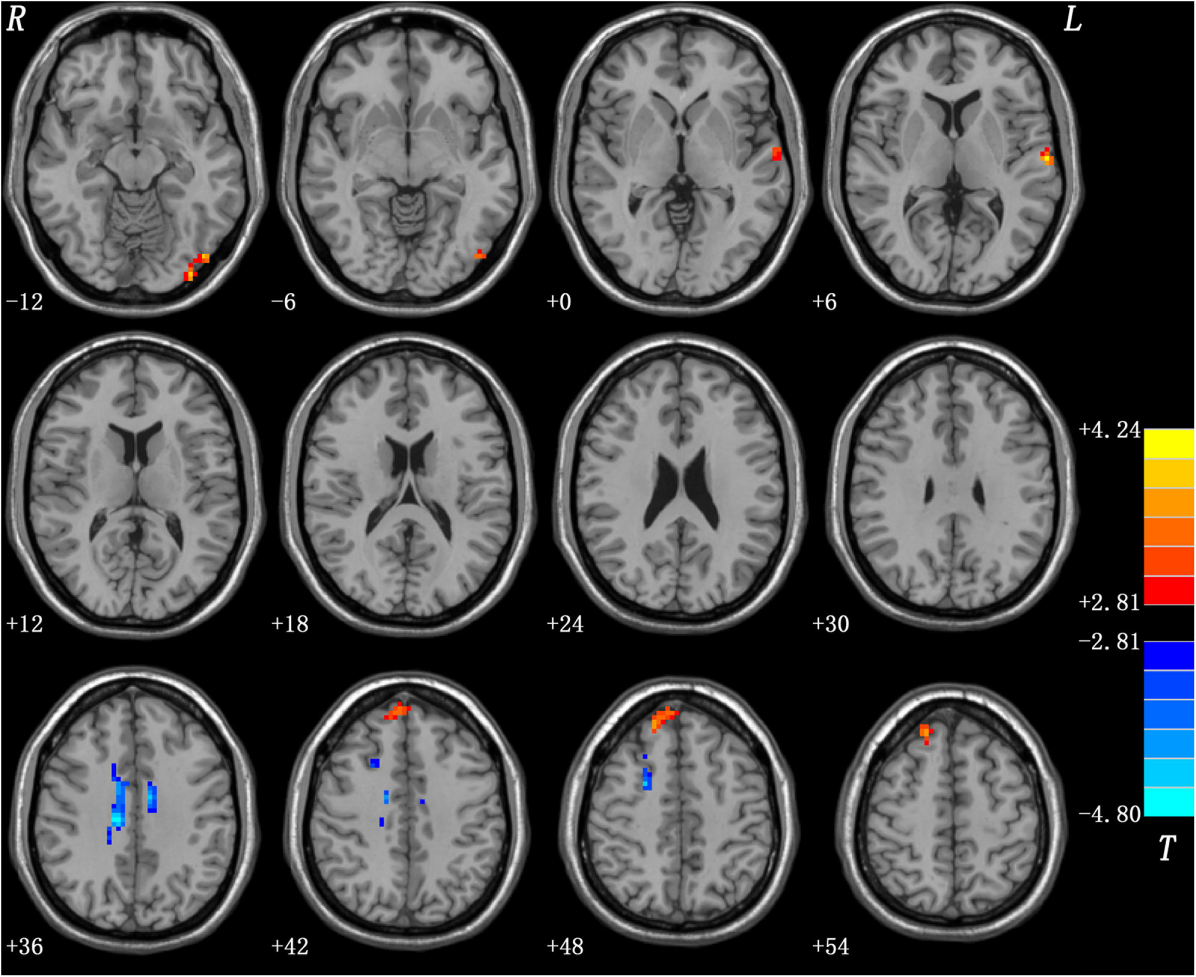
Regions with abnormal ReHo in patients with DED. Red and blue denote increased and decreased ReHo in patients with DED, respectively. DED, dry eye disease; ReHo, regional homogeneity.

Relative to the HCs, the patients with DED showed significantly enhanced ReHo in the left inferior occipital gyrus (IOG), left superior temporal gyrus (STG), and right superior medial prefrontal cortex (MPFC), and significantly reduced ReHo in the right superior frontal gyrus (SFG)/middle frontal gyrus (MFG) and bilateral middle cingulum (MC). Detailed information is presented in Table 2 and Figure 1.

**Table 2:** Regions with abnormal ReHo values in patients.

**Cluster location**	**Peak (MNI)**			**Number of voxels**	***T*-value**
	**x**	**y**	**z**		
Left inferior occipital gyrus	−45	−81	−12	36	3.8793
Left superior temporal gyrus	−63	−15	6	30	4.2411
Right superior MPFC	18	39	51	79	3.9433
Right superior frontal gyrus/middle frontal gyrus	24	3	48	30	−4.4578
Right middle cingulum	12	−21	36	84	−4.8042
Left middle cingulum	−9	−6	36	33	−4.1881

*ReHo, regional homogeneity; MNI, Montreal Neurological Institute; MPFC, medial prefrontal cortex*.

### Correlation Analysis Result

No correlation was observed between ReHo values and illness duration in patients with DED at the *P* < 0.05 level.

### SVM Result

**Figure 2:**
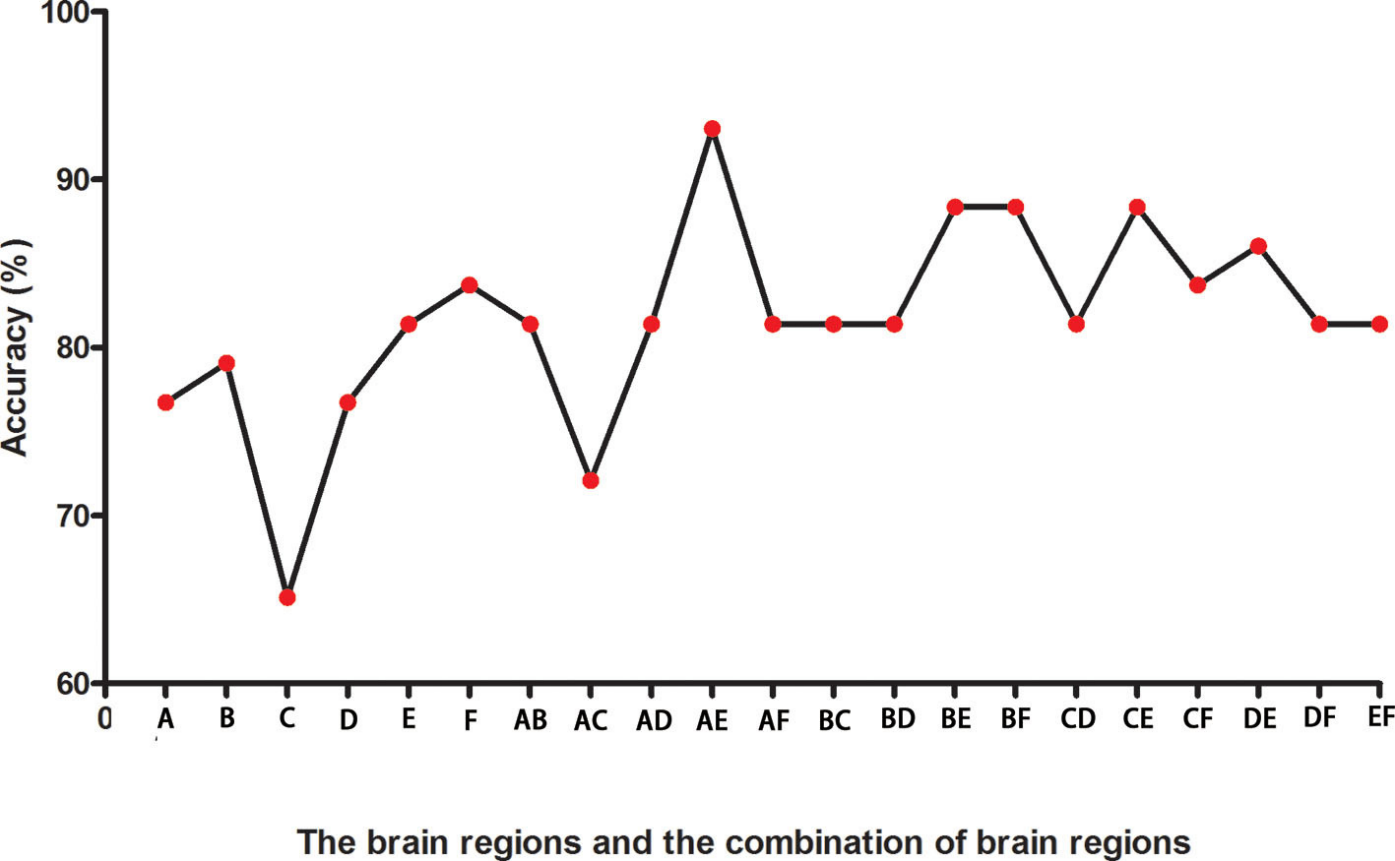
The accuracies in differentiating patients from controls of six brain regions with abnormal ReHo values and combinations of them. A represents the left middle cingulum; B represents the right middle cingulum; C represents the right superior MPFC; D represents the right superior frontal gyrus/middle frontal gyrus; E represents the left inferior occipital gyrus; F represents the left superior temporal gyrus. ReHo, regional homogeneity; MPFC, medial prefrontal cortex.

**Figure 3:**
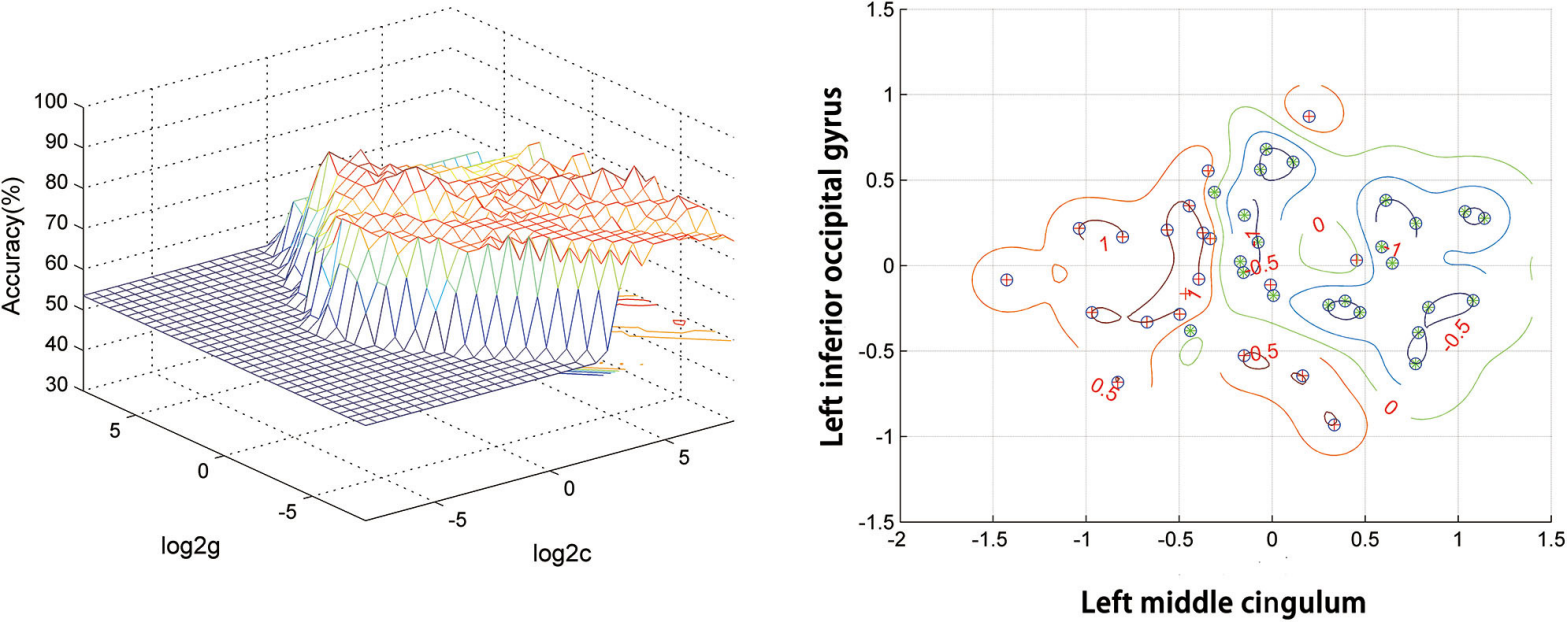
SVM analysis of the combination of ReHo values in the left middle cingulum and the left inferior occipital gyrus. Sensitivity = 95.00%, specificity = 91.30%, and accuracy = 93.02%. SVM, support vector machines; ReHo, regional homogeneity.

Figure 2 presents the accuracies for distinguishing patients with DED from HCs based on the ReHo values of six detected brain regions and a combination of these clusters. In the combination of ReHo values in the left MC and left IOG, 40 subjects were correctly classified with the highest accuracy. This combination was the optimal combination with a sensitivity of 95.00%, a specificity of 91.30%, and an accuracy of 93.02% (Figure 3).

## Discussion

Relative to the HCs, the patients with DED showed abnormal ReHo in the limbic-cortical circuits. Increased or decreased ReHo values in the patients with DED, relative to the HCs, indicated spontaneous brain activities in certain regions having more or less synchronization. No correlation was found between the ReHo values in these brain regions and illness duration in the patients. Moreover, the SVM analysis showed that a combination of ReHo values in the left MC and left IOG could facilitate the differentiation of the patients with DED from the HCs with satisfactory sensitivities, specificities, and accuracies.

### Increased ReHo Values in Left IOG, Left STG, and Right Superior MPFC

The occipital gyrus is the visual cortex, a crucial brain region for visual processing. The IOG plays a critical role in visual processing, particularly in visual processing of faces, and is known as the occipital face area. In neuroimaging studies, participants showed more activity in the IOG when observing faces than when observing other stimuli (Sergent et al., 1992; Liu et al., 2010). A damaged IOG results in the impaired identity recognition of faces (Allison et al., 1994; Rossion et al., 2003). The electrical stimulation of the IOG induces impairments in the perception of facial features and configurations (Pitcher et al., 2007; Jonas et al., 2012). Moreover, the IOG has connections to the limbic system, and a number of studies have shown neural networks consisting of the amygdala and the IOG being responsible for the visual processing of faces (Rossion et al., 2011; Sato et al., 2017). In the present study, we found significantly increased ReHo in the left IOG of the patients with DED relative to the HCs, and this result indicated reinforced activities in this brain area. Reinforced activities in the left IOG enhance visual processing. DED impairs vision, especially functional vision, in patients (Miljanović et al., 2007). Hence, increased ReHo values in the left IOG may compensate for visual impairment in patients with DED.

Repetitive transcranial magnetic stimulation creating “virtual lesions” in the STG results in disturbed visual search (Ellison et al., 2004). Another study applied intraoperative electrical stimulation and found that the inactivation of the STG induces a disturbed visual search (Gharabaghi et al., 2006). Event-related potential (Reale et al., 2007) and fMRI (Robins et al., 2009) studies have suggested that the STG plays an important role in the integration of auditory—visual cues. The STG has connections to the limbic system. The connection between the amygdala and the STG may be correlated with auditory input and the transfer of complex sensory information (Kosmal et al., 1997). Small STG and amygdala were found in patients with schizophrenia relative to HCs, and these features may be correlated with auditory hallucinations (Barta et al., 1990; Yoshida et al., 2009). In the present study, we found increased ReHo values in the left STG of the patients with DED. Therefore, increased ReHo values in the left STG may also account for visual impairment in patients with DED.

MPFC plays a crucial role in fear extinction (Milad and Quirk, 2002; Milad et al., 2004; Santini et al., 2004). A rat under persistent stress shows structural and functional changes in the MPFC (Radley et al., 2006, 2008). The MPFC is a part of the brain's reward system, and stimulating it can induce an antidepressant effect (Tzschentke, 2000; Hamani et al., 2010). The MPFC has connections to the limbic system. In these connections, the amygdala–MPFC circuit is involved in emotional processing (Delli Pizzi et al., 2017; Thijssen et al., 2017). In the present study, we found significantly increased ReHo in the right superior MPFC in the patients with DED. Patients with DED often complain of negative moods, such as depression or anxiety (Li et al., 2011; van der Vaart et al., 2015). Therefore, significantly increased ReHo in the right superior MPFC may be correlated with a negative mood in patients with DED, although anxiety and depression severity were not assessed in the present study.

### Decreased ReHo Values in the Right SFG/MFG and Bilateral MC

The right SFG generally plays a crucial role in cognitive control and emotion regulation (Rose et al., 2011; Tully et al., 2014; McDonald et al., 2020). The SFG is also involved in the experience of pain. Fulbright et al. found that the SFG, especially the right SFG, shows pain-related activation when an individual experiences pain stimuli (Fulbright et al., 2001). The frontal cortex and cingulum are parts of the “pain matrix,” which transforms nociceptive signals into a perception of pain and perceived pain intensity (Rainville, 2002; Tracey and Mantyh, 2007; Legrain et al., 2011; Favilla et al., 2014). The MFG and SFG play a vital role in modulating the nociceptive pathways of the cortical and subcortical regions (Yang et al., 2013). Decreased gray matter volume in the MFG (Absinta et al., 2012; Yang et al., 2013) and SFG (Lutz et al., 2008) was observed in painful diseases, such as cluster headaches and fibromyalgia. In the present study, we found significantly decreased ReHo values in the right SFG and MFG in the patients with DED relative to the HCs. In a majority of the patients with DED, ocular pain was a major discomfort. Hence, the dysfunction of the right SFG and MFG may be related to pain in patients with DED.

Mazzola et al. found that the stimulation of the insular cortex can evoke a pain sensation around the eye (Mazzola et al., 2006). A number of studies have found that ocular neurons have projections to the posterior thalamus and the parabrachial area and not to the main somatosensory thalamic areas (Meng et al., 1997; Aicher et al., 2013). Connections exist across the insular cortex, amygdala, and cingulate cortex, thereby suggesting the crucial role of the cerebral limbic system in the autonomic aspects of pain and affection of patients with DED (Bernard et al., 1996). The cingulate cortex is a critical region of the “pain matrix” (Favilla et al., 2014). The MC is a part of the limbic system, which is involved in emotional processing when an individual experiences pain (Zubieta et al., 2003). In the present study, we found significantly decreased ReHo values in the bilateral MC of the patients with DED relative to the HCs. In sum, the decreased ReHo values of the bilateral MC may be related to pain in patients with DED.

In the present study, we found significantly increased ReHo values in the left IOG and left STG, both of which may compensate for visual impairment in patients with DED. Increased ReHo values were also noted in the right superior MPFC, and they may be correlated with a negative mood in patients with DED. The IOG, STG, and MPFC have strong connections to the amygdala. We also found significantly decreased ReHo values in the right SFG/MFG and bilateral MC, and they may be related to the pain of patients with DED; all these four brain regions together make up the “pain matrix.” The amygdala and cingulate gyrus are part of the limbic system. The cerebral limbic system has a key role in the autonomic aspects of pain of DED patients (Bernard et al., 1996). Hence, the abnormal ReHo values found in these brain regions of the patients with DED suggested that limbic-cortical circuits may play a crucial role in the pathophysiology of DED. The dysfunction of the limbic-cortical circuits and DED symptoms may be reciprocal, and the heterogeneity of clinically observed DED symptoms can be explained by the dysfunction of the limbic-cortical circuits in combination with active intrinsic compensatory processes.

A previous study suggested that a specificity or sensitivity of more than 0.7 could be considered as an acceptable level for establishing a diagnostic index (Swets, 1988), whereas a specificity or sensitivity of <0.7 might lead to poor accuracy as a diagnostic indicator (Gong et al., 2011; Zhu et al., 2018; Li et al., 2019). In the present study, the SVM result showed that the combination of ReHo values in the left MC and left IOG in distinguishing patients with DED from HCs had a sensitivity of 95.00%, a specificity of 91.30%, and an accuracy of 93.02%. Therefore, this combination of ReHo values is appropriately applied as a potential image biomarker to distinguish patients with DED from HCs. In the correlation analysis, no correlation was observed between the ReHo values and illness duration of the patients with DED. This result suggested that the illness duration had no effect on the ReHo values.

Several limitations of the present study should be raised. The sample size is small, and it may lead to low-reliability results. Moreover, we cannot divide patients into different subgroups due to the small sample size. Large sample size studies are needed. The MNI template used in the present study was produced from a Caucasian population, and the results may be biased to Chinese subjects.

## Conclusion

In the present study, patients with DED had abnormal ReHo values in the limbic-cortical circuits. A combination of ReHo values in the left MC and the left IOG could be applied as potential imaging biomarkers to distinguish patients with DED from HCs. The dysfunction of the limbic-cortical circuits may play an important role in the pathophysiology of DED.

## Data Availability Statement

The raw data supporting the conclusions of this article will be made available by the authors, without undue reservation.

## Ethics Statement

The studies involving human participants were reviewed and approved by the ethics committee of the First Affiliated Hospital of Guangxi Medical University. The patients/participants provided their written informed consent to participate in this study.

## Author Contributions

WG and SL designed the study. SW, FL, WL, YL, XS, and HY collected the original imaging data. WG and FL analyzed the imaging data. WG, XS, and HY wrote the manuscript. All the authors contributed and approved the final manuscript.

## Conflict of Interest

The authors declare that the research was conducted in the absence of any commercial or financial relationships that could be construed as a potential conflict of interest.
